# Cerebellar transcranial static magnetic field stimulation reduces muscle activity during maximum contraction

**DOI:** 10.1186/s13104-026-07673-1

**Published:** 2026-01-25

**Authors:** Akiyoshi Matsugi, Yohei Okada, Nobuhiko Mori, Koichi Hosomi

**Affiliations:** 1 Faculty of Rehabilitation, Shijonawate Gakuen University, Hojo 5-11-10, Daitou, Osaka 574-0011 Japan; 2 Graduate School of Health Sciences, Kio University, 4-2-2 Umami-naka, Koryo-cho, Kitakatsuragi-gun, Nara, 635-0832 Japan; 3 Department of Neurosurgery, The University of Osaka Graduate School of Medicine, Yamadaoka 2-2, Suita, Osaka 565-0871 Japan

**Keywords:** Transcranial static magnetic field stimulation, Cerebellum, Maximum voluntary contraction, Resting motor threshold, Cerebellar brain inhibition, Noninvasive brain stimulation

## Abstract

**Background:**

Transcranial static magnetic field stimulation (tSMS) is a noninvasive technique that modulates neuronal excitability without inducing an electrical current. Although triple-magnet tSMS has been shown to suppress cortical excitability in the motor cortex, its effects on cerebellar circuits and motor output remain unclear.

**Objective:**

We examined whether cerebellar triple-magnet tSMS modulates voluntary muscle activation, cortical excitability, cerebellar output in healthy adults.

**Methods:**

In this single-blind, randomized, sham-controlled study, 44 healthy participants received real- or sham-tSMS over the right cerebellum. Motor output was assessed by electromyography (EMG) of the right first dorsal interosseous muscle during maximum voluntary contraction (MVC). The resting motor threshold (rMT) and cerebellar brain inhibition (CBI) were measured using transcranial magnetic stimulation.

**Results:**

EMG activity during MVC decreased in both groups, with a greater reduction in the real-tSMS group (*p* = 0.008, *r* = 0.402). No significant effect of tSMS was observed on the rMT or CBI.

**Conclusion:**

Cerebellar triple-magnet tSMS reduced EMG activity during MVC, suggesting modulation of voluntary motor control. The absence of changes in rMT and CBI suggests that tSMS may act via mechanisms not captured by conventional physiological indices.

*Trial registration* UMIN Clinical Trials Registry UMIN000058833 Date of registration 19 August 2025. Retrospectively registered.

**Supplementary Information:**

The online version contains supplementary material available at 10.1186/s13104-026-07673-1.

## Introduction

Transcranial static magnetic field stimulation (tSMS) is a noninvasive technique that modulates neuronal excitability without inducing an electrical current [1]. It has been shown to transiently suppress corticospinal excitability in the motor cortex, likely through itseffects on membrane properties and inhibitory circuits [2]. Multiple areas of the cerebral cortex, including the motor cortex, are targeted for stimulation, and clinical trials are underway to verify the effectiveness of rehabilitation [3]. Recently, a triple-magnet tSMS device [4] was developed to enhance field strength and penetration, enabling the stimulation of deeper brain structures, such as the cerebellum.

The cerebellum is crucial for motor coordination and force regulation partly through inhibitory projections to the primary motor cortex, a mechanism known as cerebellar brain inhibition (CBI) [5]. Although early tSMS studies primarily targeted cortical areas, our previous pilot study showed that single-magnet tSMS could transiently reduce CBI [6], providing initial evidence of cerebellar modulation. However, its effect on motor behavior remains unclear owing to the limited stimulation depth.

This study used a triple-magnet tSMS system to investigate whether cerebellar stimulation modulated corticospinal excitability (indexed by resting motor threshold [rMT]), cerebellar output (indexed by CBI), and voluntary muscle activation (indexed by surface electromyography [EMG] during maximum voluntary contraction [MVC]). We employed a single-blind, sham-controlled design to test the effects of cerebellar tSMS on neurophysiological and behavioral outcomes in healthy adults.

## Methods

### Participants

**Fig. 1:**
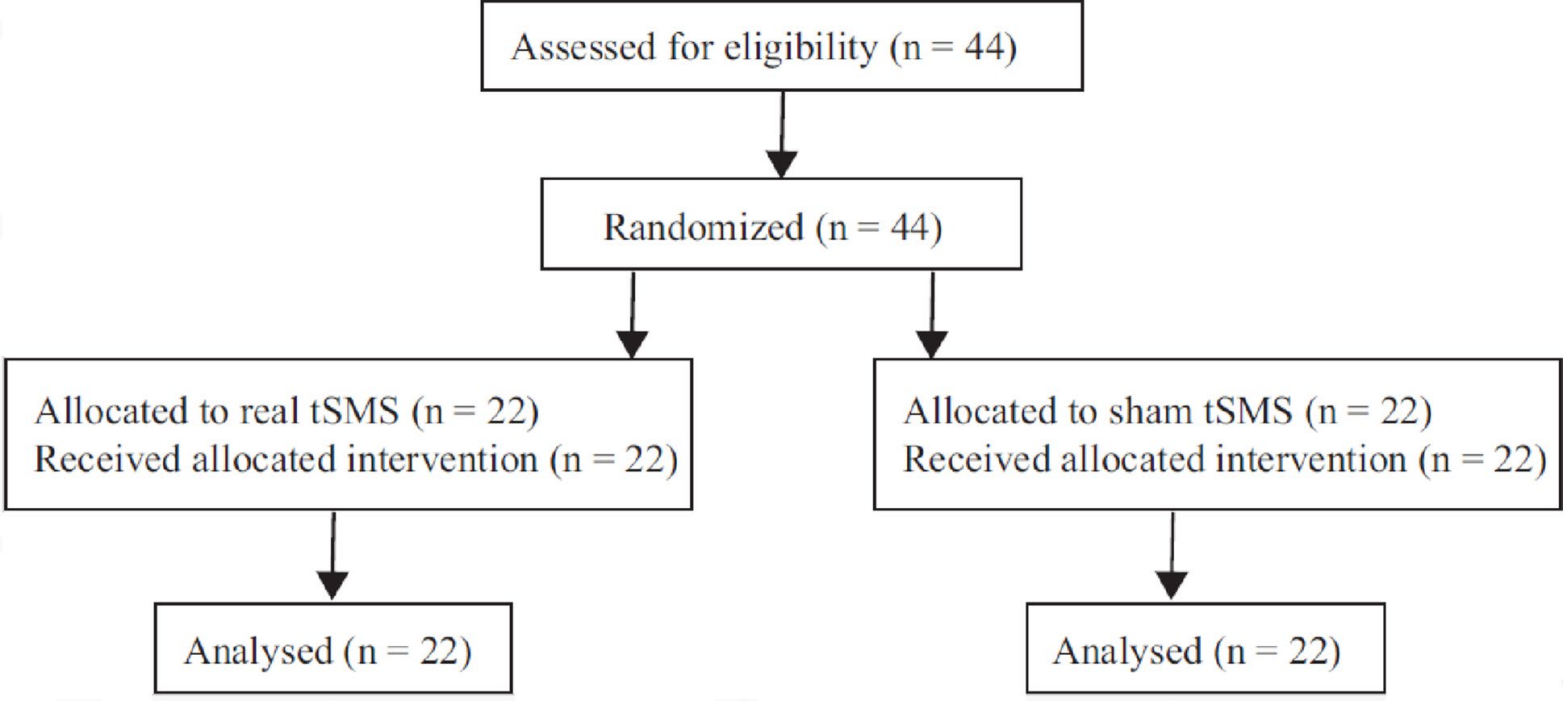
CONSORT flow diagram of participants. A total of 44 healthy adults were assessed for eligibility and randomly assigned to either the real tSMS group (*n* = 22) or the sham tSMS group (*n* = 22). All participants received the allocated intervention and were included in the final analysis. No participants were lost to follow-up or excluded after randomization

Forty-four healthy adults (mean age: 20.3 ± 0.9 years; 10 women, 34 men) were randomly assigned to either the real-tSMS (*n* = 22) or sham-tSMS group (*n* = 22). The sample size was estimated using G*Power (v3.1.9.6), assuming a large effect size (Cohen’s d = 0.8) based on a previous tSMS study [4]. Fifteen participants per group were deemed sufficient and 44 participants were recruited to ensure robustness. The CBI was included as a physiological outcome because of its good reliability and low error rate (approximately 15%) [7]. This trial was registered with the UMIN Clinical Trials Registry (UMIN000058833) on 15 August 2025, after the enrollment of participants had begun (retrospectively registered). The flow of participants through the trial is shown in Fig. 1.

### General procedure

**Fig. 2:**
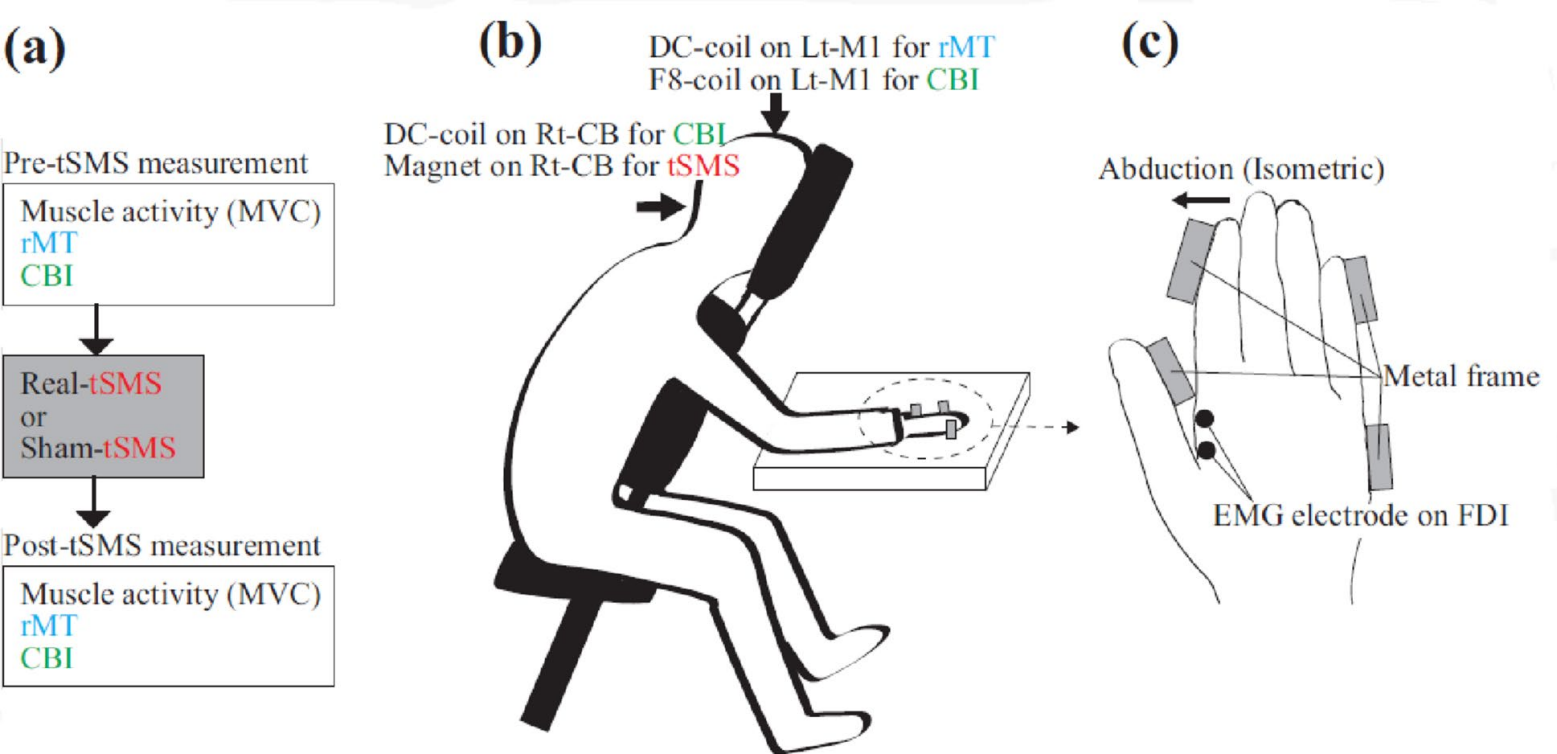
Experimental timeline and setup. **a** Experimental timeline. The transcranial static magnetic stimulation (tSMS) device (real or sham) was manually positioned over the right cerebellum for 10 min. Before and after tSMS, three trials of maximum voluntary isometric contraction (MVC) were performed and recorded the EMG activity from first dorsal interosseous (FDI) muscle, followed by the assessment of resting motor threshold (rMT) and cerebellar brain inhibition (CBI) under identical conditions. **b** Experimental setup of posture. The participant sat on a chair with a forward-leaning posture and the right forearm secured to a custom-built apparatus. For measuring rMT, a double-cone coil (DC-coil) was placed over the left primary motor cortex (M1). For measuring CBI, a figure-of-eight coil (F8-coil) was positioned over the left M1 and DC-coil was placed over the right cerebellum (Rt-CB) (1 cm below and 3 cm to the right of the inion). Paired-pulse TMS for CBI was delivered by stimulating M1 alone or both M1 and the cerebellum. To deliver tSMS, triple-magnet was placed on the Rt-CB. The same seated posture was maintained during the application of tSMS. **c** Close-up of the right-hand fixation during isometric contraction. The participant abducted the right index finger with maximal effort for 3 s while the EMG activity was recorded from the right first dorsal interosseous (FDI) muscle. This hand posture was maintained throughout MVC measurements, rMT assessment, and CBI testing.

Figure 2a shows the timeline of this experiment, including the sequence of EMG, rMT, and CBI assessments before and after tSMS. The experiments were conducted in laboratory settings. The participants were seated with their right forearm secured to a custom-built hand-fixation apparatus [8] (Fig. 2b and c). The index finger was positioned for isometric abduction. Surface EMG electrodes (Ag/AgCl) were placed 2 cm above the right first dorsal interosseous (FDI) muscle. A reference electrode was placed on the ulnar styloid. MVC, rMT, and CBI were assessed in a fixed order before and after stimulation. Photographs of the experimental setup are provided in Supplementary Figure S1

### tSMS protocol

Stimulation was delivered using a triple-magnet tSMS device (“SHIN jiba”) composed of three neodymium magnets (Model N-50; New Mag, Chiba, Japan) embedded in a nonmagnetic base (140-mm diameter, 48-mm thickness) with 16.5° inward tilt [4]. Each magnet was 50-mm diameter × 30-mm thickness, with 5340 G surface flux and 406 kJ/m³ energy density. The magnetic field characteristics of the triple-magnet device have been reported previously using simulation (ICBM152) and gaussmeter measurements, demonstrating sufficient field strength up to 80 mm depth [4] (see Supplementary Fig. S2). The device was positioned 1 cm below and 3 cm to the right of the inion to target the right cerebellum [6]. The sham condition used a visually identical nonmagnetic device [4]. Manual stimulation was performed for 10 min. The participants were blinded to the experimental conditions.

### EMG recording and MVC task

EMG signals were amplified using an MEG-1200 (Nihon Kohden, Japan), band-pass filtered (15 Hz–3 kHz), and digitized at 10 kHz using a PowerLab 800 S (AD Instruments, Australia) [9]. Participants performed three 3-s MVC trials in response to “Go” and “Stop” verbal cues, with ~ 15 s rest intervals. They were instructed to abduct their index finger maximally without visual feedback. The same posture was maintained for all measurements.

### rMT assessment

To determine rMT, a double-cone coil (D-B-80; MagVenture, Farum, Denmark) connected to a MagPro R20 stimulator (MagVenture) was placed over the left M1 [10]. The optimal site for evoking motor-evoked potentials (MEPs) in the right FDI was identified and marked. rMT was defined as the lowest intensity that elicited ≥ 50 µV MEPs in at least 50% of trials. The participants were instructed to remain relaxed, and the ongoing EMG was visually monitored to ensure no prestimulus activity.

### CBI assessment

CBI was assessed using a paired-pulse TMS protocol as described in our previous study [10]. Test MEPs were evoked using a figure-eight coil (YM-132B; Nihon Kohden, Tokyo, Japan) connected to an SMN-1200 stimulator (Nihon Kohden). The test stimulus intensity was set to produce MEPs 0.5–1 mV and was applied over the left M1. For conditioning, the same D-B-80 coil and MagPro R20 were used over the right cerebellum (1 cm below and 3 cm right of the inion) at 90% of rMT [10]. The inter-stimulus interval was 5 ms [5, 7]. Each session included both conditioned and unconditioned trials, and the same parameters were used before and after stimulation. The CBI was quantified as the natural logarithm of the ratio of conditioned to unconditioned MEP amplitudes, calculated within the predefined MEP latency window.

### Data analysis

For EMG, the root mean square (RMS) amplitude was calculated for a 1-s window at the center of the MVC trial. The mean of three trials represents each participant’s value. The EMG change was expressed as the natural log of the post/pre-RMS ratio (LN-EMG change). Change scores (delta-rMT = post-pre) were computed for rMT. For the CBI, we used the natural log of the conditioned/unconditioned MEP ratio.

Normality was evaluated using the Shapiro–Wilk test. As most variables were non-normally distributed, nonparametric tests were applied. For LN-EMG and rMT, within-group changes were tested using one-sample Wilcoxon signed-rank tests against 0 on the change scores (post–pre), and between-group differences in change scores were tested using the Mann–Whitney U test. For CBI, conditioned and unconditioned MEP amplitudes were expressed as LN(conditioned/unconditioned) [LN_C/NC]. Planned CBI analyses included within-group pre–post comparisons (Wilcoxon signed-rank test) and between-group comparisons at each time point (Mann–Whitney U test). Bonferroni correction was applied across these four planned CBI comparisons (*n* = 4), and Bonferroni-adjusted p-values are reported in the main text. In addition, as an elicitation/presence check, one-sample Wilcoxon tests against 0 were performed for LN_C/NC at each time point in each group (Real/Sham × Pre/Post; 4 tests); raw and Bonferroni-adjusted p-values for these QC tests are provided in Supplementary Table S1. Effect sizes were calculated using the rank-biserial correlation (r). For rMT, Glass’s delta was also reported using the sham group SD. In addition to the planned between- and within-group comparisons, one-sample tests against zero were conducted as an elicitation check to confirm the presence of modulation. All statistical analyses were performed using R v4.5.1 and RStudio v2025.05.1 + 513 (Posit Software), with the stats and rstatix packages.

This study was conducted and reported in accordance with the CONSORT 2025 guidelines [11]. A completed CONSORT checklist is provided as an additional file.

## Results

All 44 participants who were enrolled completed the intervention and were included in the final analysis. No participants were lost to follow-up or excluded after randomization (Fig. 1).

**Fig. 3:**
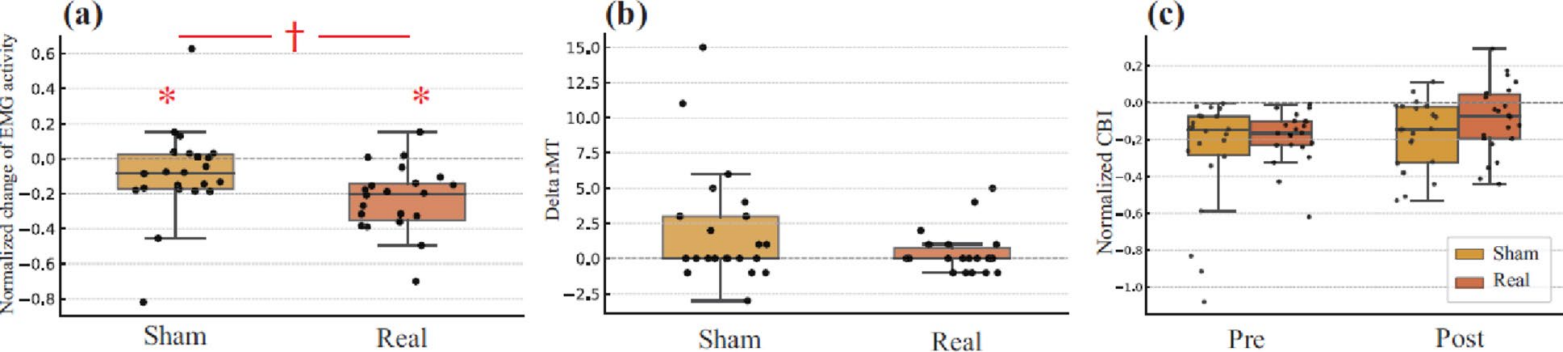
Effects of cerebellar tSMS on EMG activity, rMT, and CBI. **a** Boxplots show the natural logarithmic ratio of post to prestimulation RMS EMG amplitude during maximum voluntary isometric contraction of the first dorsal interosseous (FDI) muscle. Both sham and real tSMS groups showed significant reductions, with a greater decrease in the real group (*p* = 0.008, Mann–Whitney U test). **b** Change in resting motor threshold (rMT) from pre to poststimulation. No significant difference was found between groups (*p* = 0.206). (c) Natural logarithm of the ratio of conditioned to unconditioned motor evoked potentials, representing cerebellar brain inhibition (CBI), at pre and poststimulation timepoints. No significant changes were observed within or between groups. In all panels, individual data points are shown with jitter, and the boxplots indicate the median and interquartile range. The dashed line indicates no change. Statistical significance is indicated as follows: * *p* < 0.05 for within-group one-sample tests against 0 (Wilcoxon signed-rank test; i.e., post/pre log-ratio differs from no change), and † *p* < 0.05 for between-group comparisons (Mann–Whitney U test). *RMS* root mean square, *EMG* electromyography, *rMT* resting motor threshold, *CBI* cerebellar brain inhibition, *MEP* motor-evoked potential

Wilcoxon signed-rank tests on ΔLN-EMG showed a significant reduction in the real group (W = 10.0, *p* = 0.0000205, *r* = 0.806) and a nominal reduction in the sham group (W = 60.0, *p* = 0.030, *r* = 0.460). The Mann–Whitney U test revealed a significantly greater reduction in the real group (U = 128.5, *p* = 0.008, *r* = 0.402) (Fig. 3a).

The delta-rMT values were non-normally distributed (real: W = 0.738, *p* = 0.00006; sham: W = 0.778, *p* = 0.00023). Group comparison showed no significant difference (U = 190.0, *p* = 0.206), with small to moderate effect sizes (*r* = 0.184; Glass’s delta = − 0.391) (Fig. 3b).

Representative averaged EMG waveforms illustrating appropriate CBI elicitation, including the temporal separation of stimulation artifacts and motor evoked potentials, are provided in Supplementary Fig. S3. Normalized CBI showed a non-normal prestimulation distribution in both groups. One-sample tests confirmed that CBI was robustly inhibitory at baseline in both groups, whereas post-stimulation CBI was more variable (Supplementary Table S1). Within-group comparisons showed no significant changes from pre to postintervention (real group, adjusted *p* = 0.422, *r* = 0.350; sham group, adjusted *p* = 0.624, *r* = 0.308). Between-group differences were not significant at any time point (pre: adjusted *p* = 1.000, *r* = 0.011; post: adjusted *p* = 0.679, *r* = 0.209) (Fig. 3c). Raw and Bonferroni-adjusted p-values for the planned CBI comparisons are provided in Supplementary Table S1. In Fig. 3a, asterisks (*) denote significant within-group one-sample differences from 0 (no change), and the dagger (†) denotes a significant between-group difference.

## Discussion

In the present study, we examined whether triple tSMS over the right cerebellum modulates voluntary motor output, corticospinal excitability, and cerebellar inhibition. EMG activity during MVC was significantly reduced after real tSMS, indicating a neuromodulatory effect on voluntary muscle activation. Conversely, the rMT and CBI showed no significant changes, indicating that conventional physiological markers may not fully capture the underlying effects.

This behavioral change may be mediated by cerebellar output pathways that are not directly reflected in rMT or CBI, such as those projecting to the red nucleus or spinal circuits [12]. The effect observed in the real tSMS group is consistent with a stimulation-specific contribution despite the potential contributions from fatigue-related or order effects. Indeed, previous studies have shown that cerebellar stimulation can modulate spinal reflex excitability, possibly through descending pathways such as the rubrospinal, vestibulospinal, or reticulospinal tracts [13]. If triple tSMS alters the excitability of the spinal motor neuron pool, electrophysiological markers such as the H-reflex or F-wave may serve as useful indices in future studies. These measures can help clarify the contribution of spinal-level mechanisms to the observed reduction in EMG activity. From a practical standpoint, the protocol was feasible using simple anatomical landmarks with a visually identical sham device to maintain participant blinding (Supplementary Fig. S1).

The lack of rMT changes aligns with previous findings showing that cerebellar stimulation rarely modulates M1 excitability [10, 14], especially with techniques such as tSMS that do not generate action potentials [15]. Similarly, CBI may have been unaffected by insufficient stimulation of relevant deep structures or the limited sensitivity of the paired-pulse protocol. The dissociation between reduced EMG activity and unchanged CBI suggests that cerebellar tSMS may influence motor output through mechanisms not fully captured by conventional paired-pulse CBI paradigms. Although group differences were not statistically significant, this dissociation warrants further exploration using refined techniques such as neuronavigation-guided TMS or TMS–EEG [16], because scalp-based anatomical landmarks may not accurately reflect individual cerebellar anatomy [17]. Our results expand the scope of tSMS from cortical to cerebellar applications and support its potential for modulating motor output.

### Limitations

This study had several limitations. First, although tSMS induced a behavioral effect, no significant changes were detected in rMT or CBI. This may reflect both the measurement timing and limited sensitivity of these physiological indices. The CBI protocol primarily reflects Purkinje cell–mediated inhibition of the dentate–thalamocortical pathway, which may not fully represent the cerebellar output. Future studies should consider incorporating more sensitive and temporally precise physiological markers. Second, the EMG activity was reduced; however, functional motor performance and coordination were not assessed. Complementary kinematic or postural tasks can clarify the behavioral relevance of the tSMS. Third, the stimulation protocol was standardized anatomically but not individually. The head shape and cerebellar anatomy vary across individuals, potentially influencing the efficacy of tSMS. Neuronavigation or imaging-guided targeting may enhance precision. Fourth, the participants were healthy young adults, which limits generalizability. Investigating clinical populations with motor dysfunction, such as cerebellar ataxia, is critical for assessing the therapeutic potential. Finally, the fixed sequence of pre- and post-measurements may have contributed to nonspecific EMG reductions. A crossover design may better control such effects; however, the washout characteristics of triple tSMS after-effects are not well established and could introduce carryover. Future studies should consider crossover designs once an adequate washout period is defined.

## Supplementary Information

Below is the link to the electronic supplementary material.

Supplementary Material 1.

Supplementary Material 2.

Supplementary Material 3.

## Data Availability

The dataset supporting this study is available in the Open Data Repository: matsugi, akiyoshi; Okada, Yohei; Mori, Nobuhiko; Hosomi, Koichi (2025), “EMG activity, resting motor threshold, and cerebellar brain inhibition in a study of cerebellar transcranial static magnetic field stimulation”, Mendeley Data, V1, doi: 10.17632/b47nrc8pzw.1.

## Abbreviations

CBI: Cerebellar brain inhibition
DC-coil: Double-cone coil
EMG: Electromyography
FDI: First dorsal interosseous
LN: Natural logarithm
MEP: Motor evoked potential
MVC: Maximum voluntary contraction
M1: Primary motor cortex
rMT: Resting motor threshold
RMS: Root mean square
tSMS: Transcranial static magnetic field stimulation
TMS: Transcranial magnetic stimulation
