# Supplementary material for: Cerebellar transcranial static magnetic field stimulation reduces muscle activity during maximum contraction
Source: BMC Res Notes. 2026 Jan 25;19:84. doi: 10.1186/s13104-026-07673-1 (PMC12914903; doi:10.1186/s13104-026-07673-1)
Supplement: Supplementary file 2 — Supplementary Material 2. [file 13104_2026_7673_MOESM2_ESM.docx]

**Supplementary Figures**


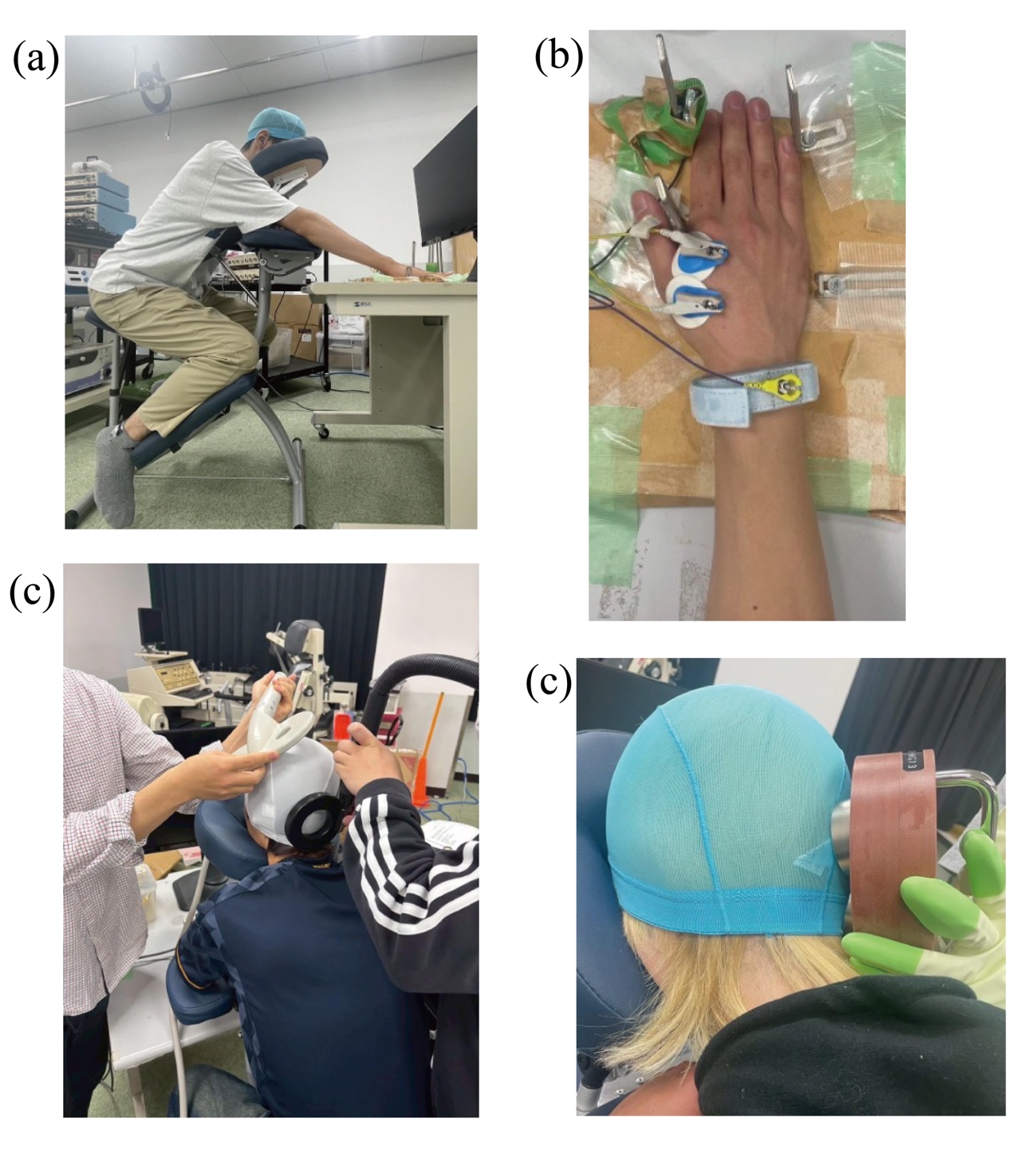


**Supplementary Figure S1. Photographs of the experimental setup.**

(a) Overall seated posture during the experiment. (b) Close-up of the right-hand fixation and EMG electrode placement on the first dorsal interosseous muscle. (c) Example of coil placement during rMT/CBI assessment. (d) Placement of the triple-magnet tSMS device over the right cerebellum (1 cm below and 3 cm to the right of the inion). Images are de-identified (no face or identifying features shown) and are published with written consent.


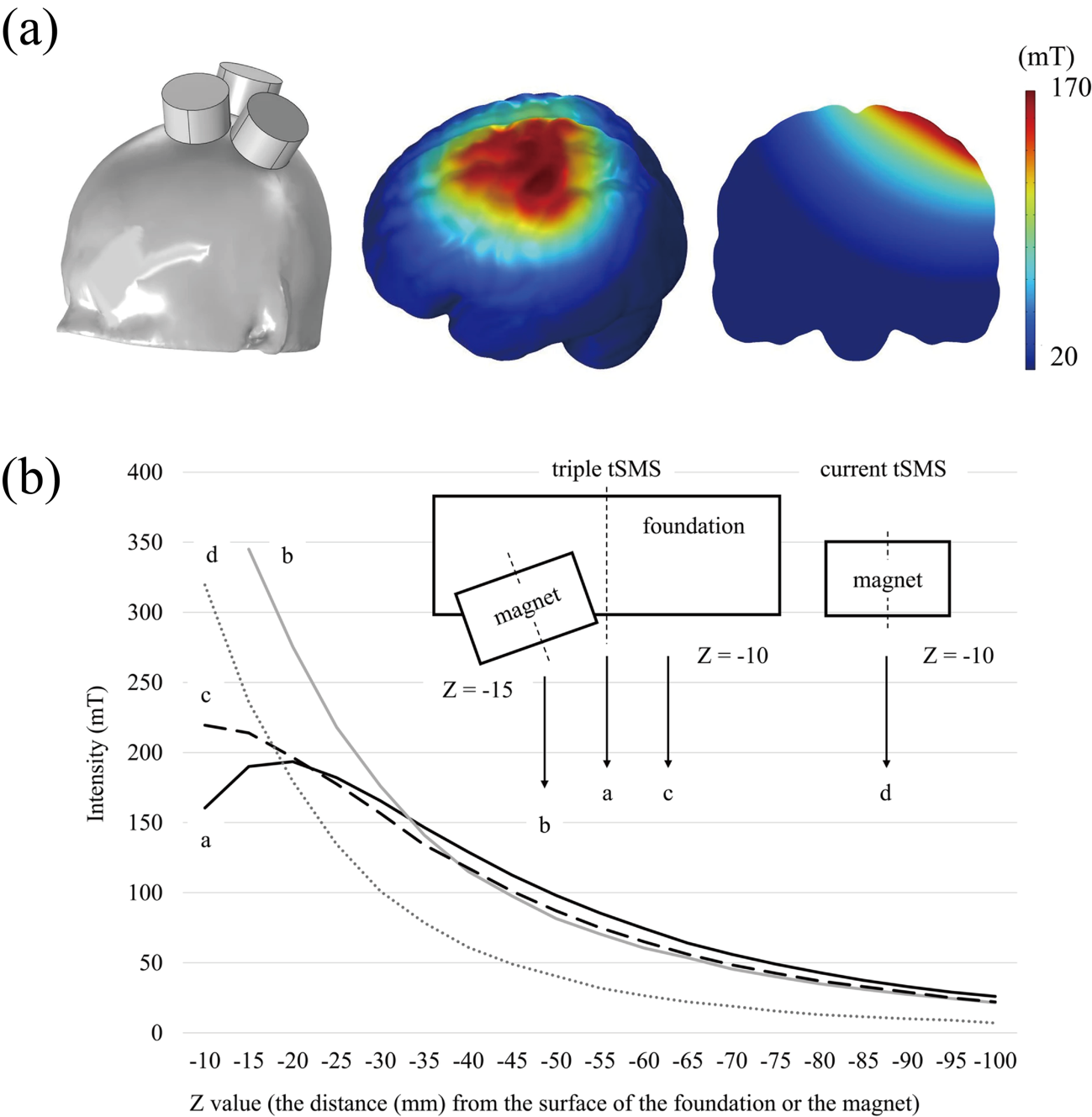


**Supplementary Figure S2. Magnetic field characteristics of the triple-magnet tSMS device (“SHIN jiba”).**

(a) Example spatial distribution of magnetic flux density simulated in a standard human brain model (ICBM152) when the device is positioned over the motor cortex. (b) Depth profile of magnetic field intensity as a function of distance (Z) from the surface of the device foundation/magnet, demonstrating slow attenuation with depth and supporting field reach to deep targets. Adapted from Shibata et al. 2022 (J NeuroEngineering Rehabil 19:129) under the Creative Commons Attribution 4.0 International License (http://creativecommons.org/licenses/by/4.0/). Changes: panel arrangement and labeling.


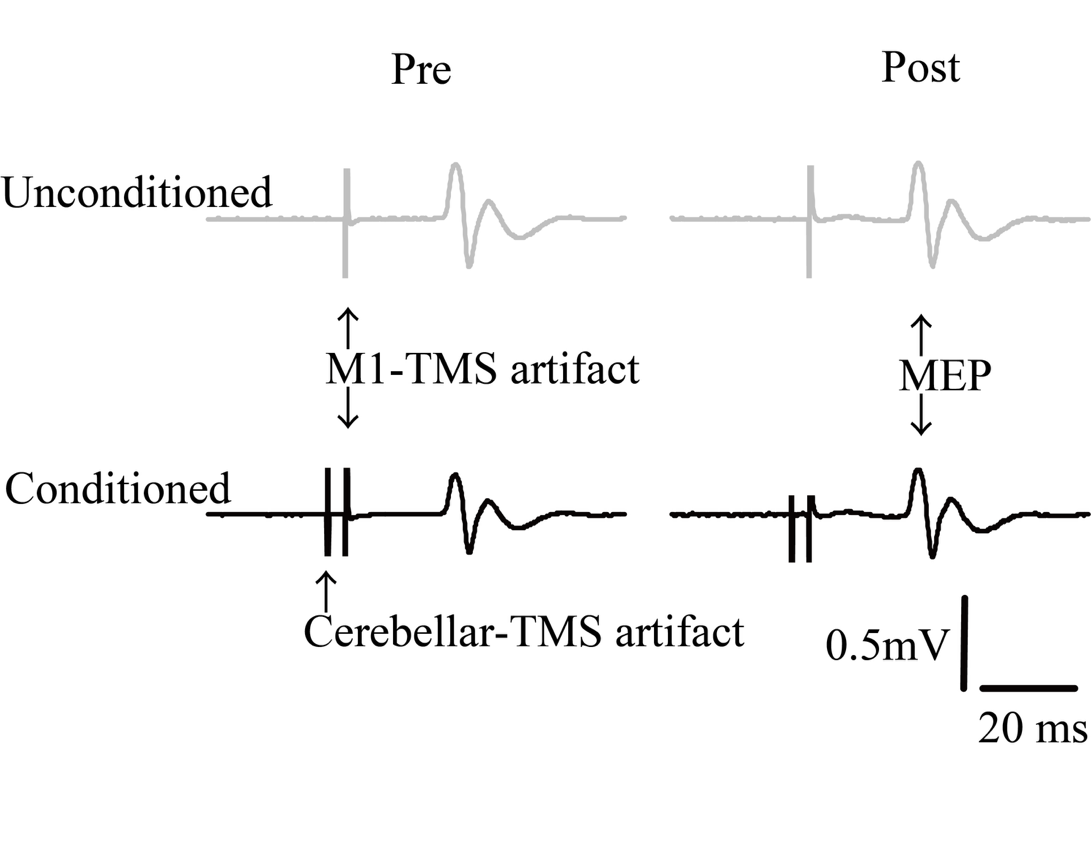


**Supplementary Figure S3. Representative averaged EMG waveforms for cerebellar brain inhibition (CBI).**

Grand-averaged EMG waveforms from all trials of a single representative participant are shown for unconditioned and conditioned stimuli before (Pre) and after (Post) stimulation. Cerebellar conditioning artifacts and M1-TMS artifacts are temporally separated from the motor evoked potentials (MEPs), which appear at the expected latency. This figure is provided for illustrative purposes.
