# Supplementary material for: Cerebellar transcranial static magnetic field stimulation reduces muscle activity during maximum contraction
Source: BMC Res Notes. 2026 Jan 25;19:84. doi: 10.1186/s13104-026-07673-1 (PMC12914903; doi:10.1186/s13104-026-07673-1)
Supplement: Supplementary file 3 — Supplementary Material 3. [file 13104_2026_7673_MOESM3_ESM.docx]

**Supplementary Table**

**A. Descriptive statistics**

| **Outcome** | **Group** | **n** | **Pre median [IQR]** | **Pre LN<0 n (%)** | **Post median [IQR]** | **Post LN<0 n (%)** |
| --- | --- | --- | --- | --- | --- | --- |
| CBI (LN_C/NC) | Real | 22 | -0.164 [-0.230, -0.100] | 22 (100%) | -0.073 [-0.194, 0.046] | 15 (68%) |
|  | Sham | 22 | -0.149 [-0.285, -0.074] | 22 (100%) | -0.145 [-0.327, -0.023] | 19 (86%) |

**B. One-sample Wilcoxon vs 0 (CBI elicitation / presence check)**

| **Time** | **Group** | **n** | **Wilcoxon W** | **raw p** | **adjusted p (Bonferroni, n=4)** |
| --- | --- | --- | --- | --- | --- |
| Pre | Real | 22 | 0 | 4.77E-07 | 1.91E-06 |
| Pre | Sham | 22 | 0 | 4.77E-07 | 1.91E-06 |
| Post | Real | 22 | 65 | 0.04616 | 0.18464 |
| Post | Sham | 22 | 16 | 8.06E-05 | 0.0003223 |

**C. Planned CBI comparisons with raw and Bonferroni-adjusted p-values**

| **Planned comparison (family n=4)** | **Test** | **Statistic** | **raw p** | **adjusted p (Bonferroni, n=4)** |
| --- | --- | --- | --- | --- |
| Within Real (Pre vs Post) | Wilcoxon signed-rank | W = 76.0 | 0.10545 | 0.42181 |
| Within Sham (Pre vs Post) | Wilcoxon signed-rank | W = 82.0 | 0.15597 | 0.62388 |
| Between groups at Pre (Real vs Sham) | Mann–Whitney U | U = 245.0 | 0.95321 | 1 |
| Between groups at Post (Real vs Sham) | Mann–Whitney U | U = 301.0 | 0.16971 | 0.67882 |

Note. CBI is expressed as LN(conditioned/unconditioned) [LN_C/NC]. Section A reports medians and interquartile ranges (IQR) and the proportion of inhibitory CBI (LN_C/NC < 0). Section B reports one-sample Wilcoxon tests against 0 as a presence/elicitation check (Real/Sham × Pre/Post; 4 tests); Bonferroni-adjusted p-values are calculated within this QC family (adjusted p = min(raw p × 4, 1.0)). Section C reports the four planned CBI comparisons (within-group pre–post in each group and between-group comparisons at pre and post; 4 comparisons), with Bonferroni adjustment applied within this planned-comparison family (adjusted p = min(raw p × 4, 1.0)).
